# Illumina Sequencing and Metabolomics Analysis Reveal Thiamine Modulation of Ruminal Microbiota and Metabolome Characteristics in Goats Fed a High-Concentrate Diet

**DOI:** 10.3389/fmicb.2021.653283

**Published:** 2021-04-07

**Authors:** Yi Ma, Chao Wang, Hao Zhang, Lihuai Yu, Li Dong, Daoqing Gong, Junhu Yao, Hongrong Wang

**Affiliations:** ^1^Laboratory of Metabolic Manipulation of Herbivorous Animal Nutrition, College of Animal Science and Technology, Yangzhou University, Yangzhou, China; ^2^School of Biomedical Sciences, The University of Western Australia, M Block, Queen Elizabeth II Medical Centre, Nedlands, WA, Australia; ^3^College of Animal Science and Technology, Northwest A & F University, Yanglin, China

**Keywords:** thiamine, SARA, 16S rRNA gene sequencing, dairy goats, LC-MS

## Abstract

Long-term supplementation of a high-concentrate diet enhances the accumulation of lactate and decrease in pH in goat rumen, thereby disrupting the composition of microbial community. Studies have shown that incorporation of thiamine in high-concentrate diet increases ruminal pH and decreases rumen lactate concentration. To explore the effects of thiamine supplementation with a high-concentrate diet on alteration of the whole ruminal microbiota and their metabolites, 18 mid-lactating Saanen goats were randomly fed with one of three diets: (1) control diet (CON; *n* = 6; concentrate:forage 30:70), (2) high-concentrate diet (HG; *n* = 6; concentrate:forage 70:30), and (3) high-concentrate diet with 200 mg of thiamine/kg of DMI (HGT; *n* = 6; concentrate:forage 70:30). The goats received experimental diets for 8 weeks. Ruminal samples were collected on the last day of the 8 weeks for 16S rRNA gene sequencing and the liquid chromatograph–mass spectrometer (LC-MS) analysis. The results revealed significant alterations of the ruminal bacterial community structure and diversity in HGT groups compared to HG groups, with an overall dominance of Bacteroidetes at the phylum level and *Oribacterium* (*P* < 0.05), *Anaerobiospirillum* (*P* < 0.01), and *Fibrobacter* (*P* < 0.01) at genus level in the HGT group. The LC-MS analysis revealed that thiamine supplementation resulted in lower levels of propionate (*P* < 0.05), pyruvate (*P* < 0.01), lactate (*P* < 0.05), putrescine (*P* < 0.05), tyramine (*P* < 0.05), and histamine (*P* < 0.01) and higher levels of acetate (*P* < 0.05), succinates (*P* < 0.01), oxaloacetic acid (*P* < 0.01), leucine (*P* < 0.01), valine (*P* < 0.05), linoleic acid (*P* < 0.05), docosahexaenoic acid (*P* < 0.05), and 4-phenylbutyric acid (*P* < 0.05) in the HGT group than in the HG group. The decrease in these compounds enhanced homeostasis in the rumen environment and suppressed epithelial inflammation. Correlation analysis revealed the potential relationships between ruminal metabolites and microbial community. These findings demonstrate that thiamine supplementation can alleviate subacute ruminal acidosis (SARA) by stabilizing the microbial community and reducing toxic unnatural compounds.

## Introduction

Subacute ruminal acidosis (SARA) is a nutritional metabolic disease that affects the finishing period of feedlot beef cattle, especially during the early and middle lactation periods of dairy cattle. The incidence of SARA is characterized by low fiber and high starch energy intake of diets, while the rumen environment exhibits no adaption to fermentation. It has been shown that short-chain fatty acids (SCFAs) and lactate generated by fermentation can be fully absorbed, thus reducing the ruminal pH, which exceeds the adaptability of animals (Kleen et al., 2003). Long-term supply of a high-concentrate diet has been found to affect the structure of the bacterial community and fermentation characteristics in the rumen (Khafipour et al., 2009; Hook et al., 2011). For instance, gram-negative bacteria die and lyse when the structure of the bacterial community changes and pH drops sharply, leading to the formation of endotoxins and histamine, which are absorbed into the blood, forming endotoxemia acidosis (Slyter, 1976; Andersen and Jarlv, 1990; Duffield et al., 2004). The absorption of histamine and lipopolysaccharide (LPS) in the rumen stimulates its epithelium to present inflammation, aggravates the damage of rumen epithelial cells, and exacerbates the condition of acidosis (Aschenbach et al., 2000; Pawlinski et al., 2003; Gressley, 2014). Recently, numerous studies have focused on the prevention of SARA. Antibiotics can control the yield of lactate by effectively inhibiting the growth of lactate-producing bacteria (Owens et al., 1998). Some studies have shown that inoculating lactate-utilizing bacteria can relieve SARA. For instance, Robinson et al. (1992) inoculated 2 × 10^12^–3 × 10^12^ *Megasphaera elsdenii* 407A in the rumen of bovine, and the concentration of lactate in the rumen decreased significantly. Elsewhere, organic acids (Callaway et al., 1997; Martin et al., 1999) and a buffering agent (Mao et al., 2016) were also applied to prevent SARA. In our previous study, we found that the supply of thiamine may serve as a novel strategy to alleviate SARA (Zhang et al., 2019). Pan et al. (2017a) opined that the supply of thiamine can stabilize the bacterial community as well as increase the pH of the rumen. Besides, the addition of thiamine could relieve the inflammatory response by decreasing endotoxin levels in the rumen and also inhibit the expression of proinflammatory cytokines in the rumen epithelium (Pan et al., 2017b; Zhang et al., 2019).

Presently, 16S rRNA sequencing technology has been widely used in agriculture, medicine, and biology to reveal microbial communities and correlations with environmental factors (Guo, 2008; Zhang H.W. et al., 2010; Zhang Y. et al., 2010; Liu et al., 2011; Patel et al., 2011; Saikia et al., 2015). Metabonomics, based on LC-MS, is an emerging tool for targeted and nontargeted analysis of small-molecule metabolites in organisms (Vuckovic, 2012; Zhao et al., 2014; Caboni et al., 2016). These key technologies have been proven valuable for further studies focusing on all aspects of molecular mechanisms (Boudonck et al., 2009; Saleem et al., 2012; Saro et al., 2018).

Thiamine is crucial to the host’s metabolism, immunity, and health. More importantly, a biologically active form of thiamine, thiamine pyrophosphate (TPP), is essential for energy metabolism (Bettendorff et al., 2014). Most of the thiamine in ruminants are derived from the synthesis of rumen microorganisms and a small part in the decomposition of feed materials (Miller et al., 1986). For ruminants, thiamine is synthesized adequately by ruminal microbes for host demand in most cases from a normal diet. Breves et al. (1981) and Miller et al. (1986) reported that ruminants require no dietary thiamine requirements because rumen microbes can synthesize it. However, Karapinar et al. (2010) and Pan et al. (2016) noted that thiamine deficiency occurred when sheep or cattle had subacute or acute ruminal acidosis, which is associated with increasing thiamine degradation by thiaminase and decreasing microbial thiamine synthesis activity under high-grain-induced SARA (Brent, 1976). Ruminants have been established to acquire subacute or acute ruminal acidosis due to a high-concentrate diet, while the exchange of substances and energy may occur abnormally, causing thiamine deficiency (Karapinar et al., 2010; Pan et al., 2016). More interestingly, thiamine supplementation when feeding high-concentrate diets can increase ruminal pH, followed by a concomitant decrease in ruminal lactate concentration (Pan et al., 2016), thereby increasing the population of ruminal cellulolytic bacteria (Pan et al., 2017a). Our previous study uncovered that thiamine supplementation with a high-concentrate diet can increase ruminal pH along with the reduction of rumen lactate concentration (Pan et al., 2016). In addition, we also have demonstrated that thiamine supplementation with a high-concentrate diet can improve the rumen epithelial activity of TPP-dependent enzymes (unpublished results). In another study, thiamine regulated the structure of the rumen microbial community of SARA cows by reducing the population of *Streptococcus bovis*, thereby promoting the growth of *M. elsdenii* (Wang et al., 2015).

Previous studies have used 16S rRNA sequencing technology to explore the correlations between the microbial communities and thiamine in dairy cows during SARA (Pan et al., 2017a). Unfortunately, there is a dearth of information regarding the relationship between the supply of thiamine and rumen metabolites. We thus hypothesize that thiamine supplementation can modulate ruminal fluid microbiota and metabolites of goats fed a high-concentrate diet.

## Materials and Methods

### Animals, Feedstuffs, and Feeding Regimes

All animal experiments were conducted in line with the Animal Protection Law based on the Guidelines for the Care and Use of Laboratory Animals approved by the Ethics Committee of Yangzhou University (SXXY 2015-0054).

Eighteen mid-lactating (148 ± 3 DIM) Saanen goats (body weight = 36.5 ± 1.99 kg; body condition score = 2.73 ± 0.16, where 0 = emaciated and 5 = obese; Russel et al., 1969) in parity 1 or 2 were used. All goats were randomly fed one of three diets: (1) control diet (CON; *n* = 6; concentrate:forage 30:70), (2) high-concentrate diet (HG; *n* = 6; concentrate:forage 70:30), (3) high-concentrate diet with 200 mg of thiamine/kg of DMI (HGT; *n* = 6; concentrate:forage 70:30). The supplementation dose of thiamine was based on previous studies on lambs (Neville et al., 2010), dairy goats (Zhang H.W. et al., 2010), and cows (Zhang et al., 2014; Pan et al., 2017b). Goats in each group were fed on the respective diets for 8 weeks. Dietary components of dairy goats were formulated according to NRC (2001). The dietary components are presented in Supplementary Table 1, and the data have been published in the *Journal of Dairy Science* (Zhang et al., 2019). The goats were fed at 7:00 and 18:00 and with one half of the allowed daily dose at each feeding. Thiamine was mixed with the concentrate to be licked by the goats. During the trial period, goats were allowed free access to freshwater.

### Collection and Measurement of Rumen Samples

On the last day of week 8, the goats were slaughtered 4 h after feeding, and a representative sample of ruminal digesta (at least 200 ml) was collected from the ruminal ventral sac. Then, 200 ml of rumen fluid was collected from each animal and transferred into a separate sterilized container, immediately frozen with liquid nitrogen, and stored at −80°C for further processing. In total, 18 rumen fluid samples were collected from the goats; however, two rumen fluid samples from the HGT group were damaged, and therefore, metabolomic analysis was only performed for the remaining 16 rumen fluid samples. A pH meter (Sartorius, Göttingen, Germany) was used to measure rumen pH using the collected samples. One hundred milliliters of the fluid samples was filtered through four layers of cheesecloth and centrifuged at 10,000 × *g* for 15 min at 4°C to obtain the supernatant, which was used to determine the concentration of volatile fatty acids (VFAs) and ammonia nitrogen (NH_3_-N). The content of thiamine in the rumen fluid was measured by high-performance liquid chromatography (HPLC) according to the Analytical Methods Committee (2000). Another 100 ml of aliquot was stored at −80°C immediately for microbial DNA extraction and metabolomic analysis.

### DNA Extraction, 16S rRNA Gene Amplification, and Sequencing

The collected rumen samples were thawed at room temperature, and a 1-ml aliquot was centrifuged at 10,000 × *g* for 1 min at 4°C, and then the supernatant was discarded. DNA was extracted with a bead-beating method using a mini-bead beater (BioSpec Products, Bartlesville, United States) as per manufacturer’s instructions, followed by phenol–chloroform extraction (Zoetendal et al., 1998). After the solution was precipitated with anhydrous ethanol, the pellets were suspended in the EDTA buffer. The DNA was determined spectrophotometrically using a Nanodrop spectrophotometer (Nyxor Biotech, Paris, France), following staining with QIAamp DNA Stool Mini Kit (Qiagen, Hilden, Germany). DNA was stored at −80°C until further processing.

Next-generation sequencing (NGS) library preparations and Illumina MiSeq sequencing were conducted at Novogene Inc. (Nanjing, China). The V3–V4 regions of the bacterial 16S rRNA gene were amplified by PCR using a forward primer (CCTACGGRRBGCASCAGKVRVGAAT) and reverse primer (GGACTACNVGGGTWTCTAATCC). These primers also contained adapter sequences that allow uniform amplification of highly complex libraries in preparation for downstream NGS on Illumina MiSeq. All PCRs were performed in a 50-μl thermal cycler (Bio-Rad, United States) containing 4 μl of fivefold FastPfu buffer, 10 ng of DNA, 0.8 μM of each primer, 0.5 U of Pfu polymerase, and 2 μl of 2.5 mM dNTPs. PCR conditions utilized for amplification were as follows: first step of 95°C for 3 min, followed by 27 cycles at 95°C for 30 s, 55°C for 30 s, 72°C for 45 s, and a final extension at 72°C for 10 min. The amplicons were purified using the AxyPrep DNA gel Extraction Kit (Axygen Biosciences, Union City, CA, United States). Thereafter, amplicon libraries were generated using a TruSeq^TM^ DNA Sample Prep Kit (TransGen Biotech, China) according to the manufacturer’s protocol. Paired-end (2 × 250 bp) sequencing was performed with the Illumina MiSeq platform based on the manufacturer’s guidelines (Caporaso et al., 2010).

### Sequence Processing and Analysis

We used Cutadapt V1.9.1 software (Martin, 2011) to trim the low-quality part of sequence reads, while sample data were extracted from the reads obtained using barcodes. Next, we removed barcode and primer sequences to obtain raw reads. Then read sequences (Rognes et al., 2016) were compared against the species annotation database to detect the chimeric sequences, which were subsequently removed (Haas et al., 2011) to obtain the final clean reads. Using UPARSE V7.0 software, clean reads from all samples were clustered (Edgar, 2013), whereby 97% of identity sequences were clustered into operational taxonomic units (OTUs). Then, sequences with the highest occurrence frequency in OTUs were screened out as the representative sequences of OTUs following the principles of the algorithm. Additionally, community richness and diversity were measured using the Chao1 and Shannon indices, respectively. Species annotation analysis was conducted using the Mothur method and SILVA132 (Wang, 2007) SSU rRNA database (Quast and Pruesse, 2013) (the threshold value was set as 0.8–1) so as to obtain taxonomic information and make statistics of the community composition of each sample at various classification levels, including kingdom, phylum, class, order, family, genus, and species. The data of each sample were homogenized. We employed QIIME 1.9.1 and R 2.15.3 software to execute different analyses between alpha diversity index groups.

### LC-MS Metabolomics Processing

A total of 16 rumen samples were analyzed using the LC-MS platform as previously reported elsewhere (Chen et al., 2009; Zhu, 2013). Afterward, samples were resuspended with prechilled methanol and 0.1% formic acid by vortexing, followed by incubation on ice for 5 min and centrifugation at 15,000 rpm at 4°C for 5 min. After centrifugation, supernatants were diluted to a final concentration of 60% methanol using LC-MS-grade water. The samples were subsequently transferred to a fresh Eppendorf tube with a 0.22-μm filter and then centrifuged at 15,000 × *g* at 4°C for 10 min. Finally, the filtrate was injected into the LC-MS system for analysis. All LC-MS analyses were performed using a Vanquish UHPLC system (Thermo Fisher) coupled with an Orbitrap Q Exactive HF-X mass spectrometer (Thermo Fisher). Briefly, samples were injected onto a Hypersil Gold column (100 × 2.1 mm, 1.9 μm) using a 16-min linear gradient at a flow rate of 0.2 ml/min. The eluents for the positive polarity mode were eluent A (0.1% FA in water) and eluent B (methanol), while those of the negative polarity mode were eluent A (5 mM ammonium acetate, pH 9.0) and eluent B (methanol). The solvent gradient were set as follows: 2% B, 1.5 min; 2–100% B, 12.0 min; 100% B, 14.0 min; 100–2% B, 14.1 min; and 2% B, 16 min. Lastly, the Q Exactive HF-X mass spectrometer was operated in positive/negative polarity mode with a spray voltage at 3.2 kV, capillary temperature at 320°C, sheath gas flow rate at 35 arb, and aux gas flow rate at 10 arb.

### Metabolomics Data Analysis

To perform peak alignment, peak picking, and quantitation for each metabolite, we processed the raw data files generated by ultra-HPLC–mass spectrometry (UHPLC-MS/MS) using the Compound Discoverer 3.0 (CD 3.0, Thermo Fisher). For data collection, the main parameters were set as follows: retention time tolerance, 0.2 min; actual mass tolerance, 5 ppm; signal intensity tolerance, 30%; signal/noise ratio, 3; and minimum intensity, 100,000. Then, peak intensities were normalized to the total spectral intensity. The normalized data were used to predict the molecular formula based on additive ions, molecular ion peaks, and fragment ions. In order to obtain accurate qualitative and quantitative results, the peaks were matched against the mzCloud and ChemSpider databases.

After Pareto scaling treatment, principal component analysis (PCA) was performed on the peaks extracted from all trial samples. The PLS-DA variable importance in the projection (VIP) values combined with *P*-values of the *t*-test were used to highlight significant differential expression metabolites. The screening criteria were as follows: threshold (VIP) > 1.0 and difference multiple (FC) > 2.0 or FC < 0.5 and *P*-value < 0.05. In addition, significantly differentially expressed metabolites were analyzed for expression pattern clustering using the gplots package in R (Warnes et al., 2016). Correlations between different metabolites and bacterial communities were explored with Spearman’s correlation analysis using the pheatmap package in R (Kolde, 2012). For all analyses, FDR-corrected *P*-values below 0.05 were considered statistically significant.

## Results

### Rumen Fermentation Parameters

The fermentation and thiamine characteristics (pH, NH3-N, VFA, LPS, and PDH profile) are detailed in Supplementary Table 2. The data have been published in the *Journal of Dairy Science* (Zhang et al., 2019). In summary, thiamine altered rumen fermentation characteristics.

### Richness, Diversity Estimates, and Rumen Bacteria Composition

**FIGURE 1:**
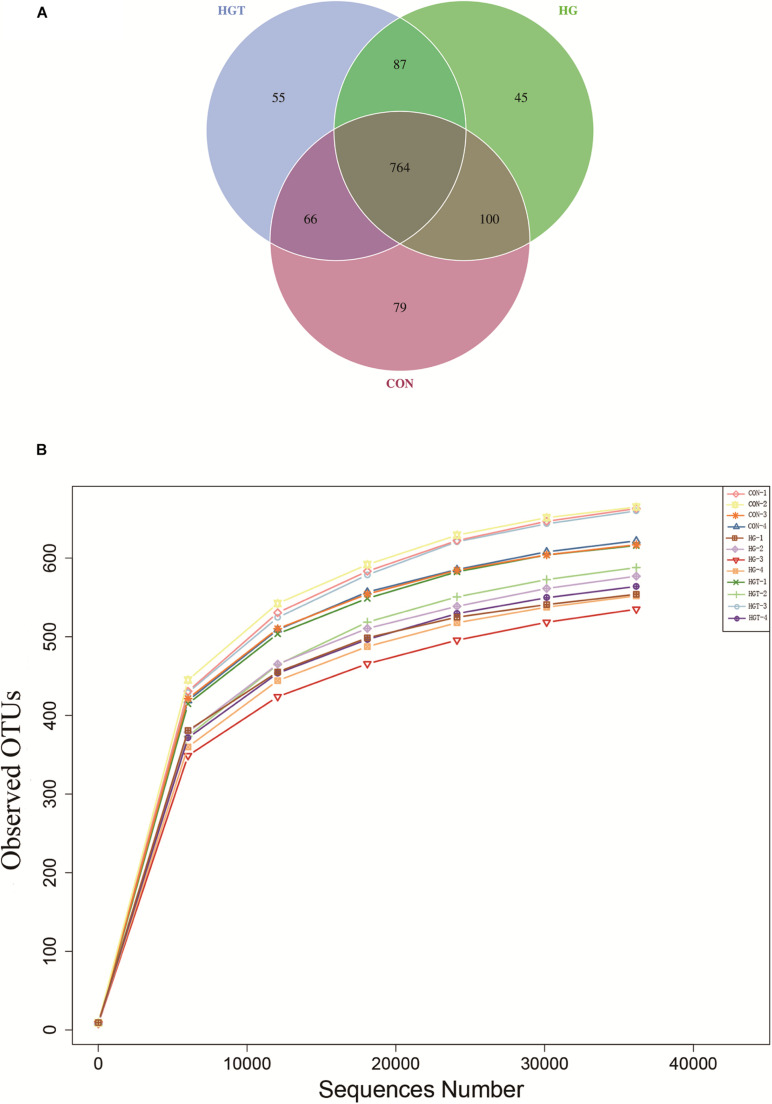
Ruminal microbial operational taxonomic units (OTUs) with different treatments of diets. CON: basal diets without supplement; HG: high-concentrate diet; HGT: high-concentrate diet supplemented with 200 mg of thiamine / kg of DMI. **(A)** Venn diagram of ruminal bacterial OTUs. The number of unique OTUs were represented by the non-overlapped portion of Venn diagram for each group. **(B)** Bacterial rarefaction curves based on OTUs were used to assess the depth of coverage for each sample.

**FIGURE 2:**
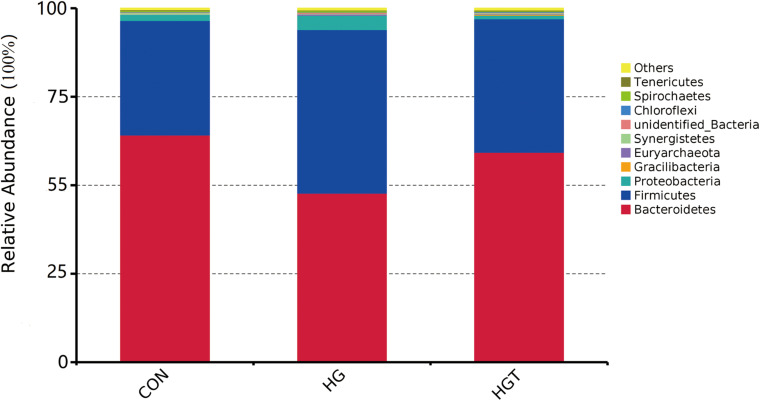
Percentage composition of the top 10 predominant phyla in rumen fluid. CON: basal diets without supplement; HG: high-concentrate diet; HGT: high-concentrate diet supplemented with 200 mg of thiamine / kg of DMI.

A total of 624,283 reads were detected from 12 samples via Illumina sequencing. Read shear filtration and quality control obtained an average of 58,551 effective data, while the quality control effective rate reached 93.88%. Sequences were clustered into OTUs at a 97% similarity level, generating a total of 1,257 OTUs. The Venn diagram shows that the intersection numbers of OTU for the CON, HG, and HGT groups were 764 (Figure 1A). Further, rarefaction curves revealed that the curve flattens out as the sequencing deepens, demonstrating that the OTU of the HGT group was higher than that of the HG group but lower than that of the CON group at the same sequencing depth (Figure 1B). Within the bacterial population, we uncovered three dominant phyla, namely, Bacteroidetes (53.41%), Firmicutes (36.32%), and Proteobacteria (3.36%) (Figure 2).

In terms of alpha bacterial diversity (Table 1), we observed no significant differences in OTU and Good’s coverage between the CON and HGT groups. Compared with the CON group, the feeding of high-grain diets (HG) markedly decreased Chao1 richness, abundance-based coverage estimator (ACE), and Shannon diversity index. Supplementation with thiamine significantly increased Shannon (*P* < 0.05), but the increases in ACE, Chao1 value, and Simpson index were insignificant (*P* > 0.05) in the HGT group compared with the HG group.

**TABLE 1:** Number of observed species, richness, and diversity indices in ruminal samples from each dietary treatment.

Items	Treatments^1^			SEM^2^	*P*-value
	CON	HG	HGT		
OTU^3^	612.50	576.25	587.50	7.75	0.147
ACE^4^	675.39^a^	628.46^b^	659.04^ab^	8.12	0.036
Chao1	705.20^a^	669.38^b^	683.90^ab^	6.06	0.032
Shannon	6.81^a^	6.71^b^	6.79^a^	0.02	0.023
Simpson	0.95	0.91	0.92	0.01	0.102
Coverage (%)	99.8	99.8	99.8	0.00	0.405

*^a,b^Means within a row with different letters differ significantly (P < 0.05).*

*^1^CON, basal diets without supplement; HG, high-concentrate diet; HGT, high-concentrate diet supplemented with 200 mg of thiamine/kg of DMI.*

*^2^SEM, standard error of the mean.*

*^3^OTU, operational taxonomic units.*

*^4^ACE, abundance-based coverage estimator.*

**FIGURE 3:**
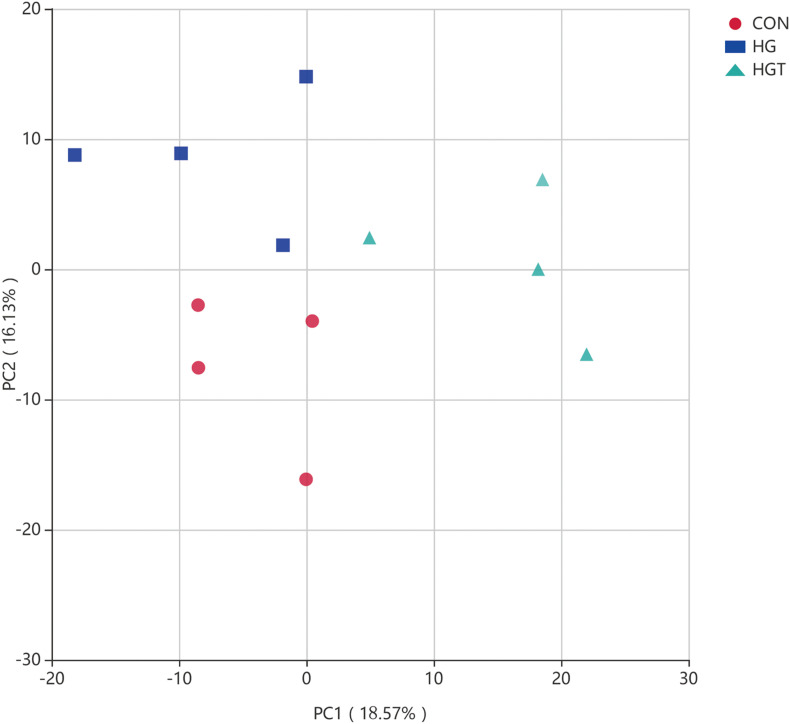
Principal Component Analysis (PCA) of bacterial community structures of the ruminal microbiota in CON (red circles), HG (blue rectangle), and HGT (green triangle) groups. CON: basal diets without supplement; HG: high-concentrate diet; HGT: high-concentrate diet supplemented with 200 mg of thiamine / kg of DMI.

PCA revealed that goats fed with HG diet were distinctly separate from goats in the CON and HGT groups (Figure 3). Importantly, principal coordinates 1 and 2 accounted for 13.57 and 11.13% of the total variation, respectively.

### Effect of Thiamine Supplementation on Relative Abundance of Bacterial Communities

At the phylum level (Table 2), the HG diet significantly decreased the relative abundance of Bacteroidetes (*P* < 0.01) and Firmicutes (*P* < 0.05), as well as increased the relative abundance of Firmicutes (*P* < 0.05) compared with the CON groups. Whereas supplementation with thiamine in the HGT group considerably increased the abundance of Bacteroidetes (*P* < 0.01), the increase of Fibrobacteres and the decrease of Firmicutes in relative abundance were insignificant (*P* > 0.05) compared with those in the CON group. We also identified no significant shifts in the relative abundance of Proteobacteria, Synergistetes, Euryarchaeota, Spirochaetes, Gracilibacteria, and Chloroflexi with the supplementation of HGT and thiamine compared with the CON group (*P* > 0.05).

**TABLE 2:** Effects of thiamine supplementation on relative abundance in rumen fluid at phylum level^1^.

Phylum	Treatments^2^			SEM^3^	*P*-value
	CON	HG	HGT		
Firmicutes	32.37^b^	46.20^a^	38.78^ab^	2.22	0.016
Bacteroidetes	64.17^a^	48.76^b^	58.30^a^	2.24	0.000
Proteobacteria	1.64	2.26	1.36	0.19	0.159
Fibrobacteres	0.11^a^	0.06^b^	0.08^b^	0.01	0.008
Synergistetes	0.05	0.05	0.03	0.00	0.248
Euryarchaeota	0.11	0.15	0.13	0.02	0.551
Spirochaetes	0.32	0.37	0.27	0.04	0.652
Gracilibacteria	0.05	0.03	0.03	0.00	0.663
Chloroflexi	0.43	0.55	0.49	0.04	0.488
unidentified_Bacteria	0.27	0.46	0.34	0.05	0.373

*^a,b^Means within a row with different letters differ significantly (P < 0.05).*

*^1^Only phylum level (relative abundance is in the top 10) that affected by treatments were listed.*

*^2^CON, basal diets without supplement; HG, high-concentrate diet; HGT, high-concentrate diet supplemented with 200 mg of thiamine/kg of DMI.*

*^3^SEM, standard error of the mean.*

At the genus level (Table 3), compared with the CON group, high-concentrate diet (HG) substantially decreased the relative abundance of *Oribacterium*, *Acetitomaculum*, *Saccharofermentans*, *Bacteroides*, uncultured *Prevotellaceae*, *Anaerobiospirillum*, *Papillibacter*, and *Fibrobacter* (*P* < 0.05) but also increased the abundance of *Succiniclasticum*, *Prevotella_1* (*P* < 0.05), and *Prevotella_7* (*P* < 0.01). While supplementation with thiamine largely increased the relative abundance of *Oribacterium*, uncultured *Prevotellaceae* (*P* < 0.05), *Anaerobiospirillum*, and *Fibrobacter* (*P* < 0.01), as well as decreased the relative abundance of *Prevotella_1* (*P* < 0.05), but the effect on the relative abundance of *Succiniclasticum*, *Acetitomaculum*, *Saccharofermentans*, *Bacteroides*, and *Prevotella_7* was insignificant compared with that of high-concentrate diet (*P* < 0.05). Notably, the results of correlation analysis revealed that phenyl propionic acid was positively associated (*r* > 0.38, *P* < 0.05) with 11 taxa and negatively associated (*r* < −0.38, *P* < 0.05) with four taxa and several ciliate and methanogens.

**TABLE 3:** Effects of thiamine supplementation on relative abundance in rumen fluid at genus level^1^.

Phylum	Family	Genus	Treatments^2^			SEM^3^	*P*-value
			CON	HG	HGT		
Firmicutes	Veillonellaceae	Succiniclasticum	3.12^b^	6.97^a^	4.83^ab^	0.60	0.013
	Lachnospiraceae	Oribacterium	0.36^ab^	0.22^b^	0.47^a^	0.04	0.037
		Acetitomaculum	0.89^a^	0.62^b^	0.76^ab^	0.04	0.012
	Ruminococcaceae	Papillibacter	0.54^a^	0.32^b^	0.24^b^	0.05	0.004
		Saccharofermentans	0.73^a^	0.49^b^	0.68^ab^	0.04	0.031
Bacteroidetes	Bacteroidaceae	Bacteroides	0.79^a^	0.47^b^	0.64^ab^	0.05	0.014
	Prevotellaceae	Prevotella_1	7.44^a^	8.17^a^	6.54^b^	0.27	0.022
		Prevotella_7	0.17^b^	0.77^a^	0.58^a^	0.08	0.000
		Uncultured Prevotellaceae	0.16^a^	0.09^b^	0.14^a^	0.01	0.015
Proteobacteria	Succinivibrionaceae	Anaerobiospirillum	0.25^b^	0.18^c^	0.34^a^	0.02	0.006
Fibrobacteres	Fibrobacteraceae	Fibrobacter	0.34^a^	0.11	0.27^a^	0.07	0.002

*^a,b^Means within a row with different letters differ significantly (P < 0.05).*

*^1^Only bacterial genera (accounted for ≥ 0.1% in at least one of the samples) that affected by treatments were listed.*

*^2^CON, basal diets without supplement; HG, high-concentrate diet; HGT, high-concentrate diet supplemented with 200 mg of thiamine/kg of DMI.*

*^3^SEM, standard error of the mean.*

### Rumen Metabolomics Profiling

#### Sample Quality Control

We further evaluated the stability and reproducibility of data using the QC samples measured during the whole experimental period. The higher the QC sample correlation (*R*^2^ close to 1), the better the quality of data. The correlation of QC samples is shown in Supplementary Figure 1. The *R*^2^ values of the positive and negative polarity modes were 0.981 and 0.986, respectively.

#### Differential Metabolite Analysis

The PCA results among the CON group, HG group, and HGT group. Remarkably, PCA provided a satisfactory separation of data among the high-concentrate diet group (HG), high-concentrate diet + thiamine group (HGT), and the CON group (Supplementary Figure 2).

**FIGURE 4:**
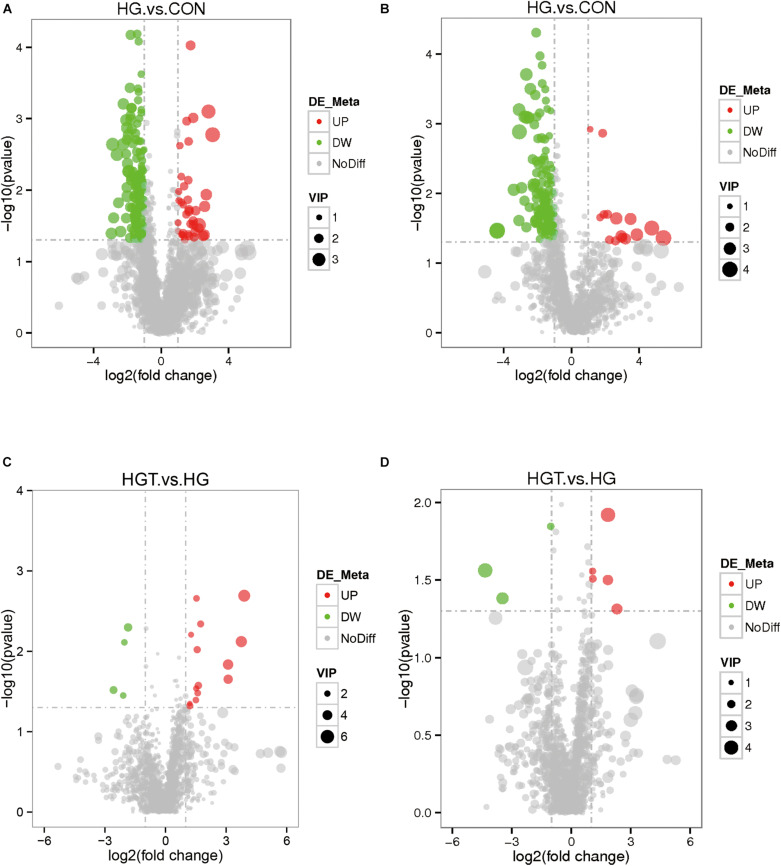
The volcano plot of differential metabolites with different treatments of diets. The gray plot shows there is no difference, the red plots show up-regulated endogenous metabolites, while the green plots show down-regulated endogenous metabolites. VIP value represents the importance projection value of the metabolite obtained in the PLS-DA model compared in this group. CON: basal diets without supplement; HG: high-concentrate diet; HGT: high-concentrate diet supplemented with 200 mg of thiamine / kg of DMI. **(A)** The volcano plot between the HG and the HGT group in the positive polarity mode. **(B)** The volcano plot between the HG and the HGT group in the negative polarity mode. **(C)** The volcano plot between the HGT and the HG group in the positive polarity mode. **(D)** The volcano plot between the HGT and the HG group in the negative polarity mode.

The volcano maps of differential metabolites. We employed volcanic diagrams to show the number of differential metabolites in each comparison. As shown in Figure 4, the gray plot indicates no difference, the red plots show upregulated endogenous metabolites, and the green plots show downregulated endogenous metabolites. There were 14 common differential metabolites between the two comparisons for the HG versus CON groups and HGT versus HG groups that presented different expression trends (Table 4).

**TABLE 4:** Different metabolites content in the rumen of goats fed SARA-induced diet (HG) versus goats fed SARA-induced diet with thiamine supplementation (HGT).

No.	Metabolites	Formula	Group^1^	VIP^2^	*P*-value	Log_2_ FC^3^	Trend
**Carbohydrate metabolism**							
1	Acetate	C_2_H_4_O_2_	HG vs CON	1.6354	*P* < 0.01	–2.0534	down
			HGT vs HG	1.3654	*P* < 0.05	2.0534	up
2	Propionate	C_3_H_6_O_2_	HG vs CON	1.4863	*P* < 0.01	1.5273	up
			HGT vs HG	1.2853	*P* < 0.05	–3.2273	down
3	Pyruvate	C_3_H_4_O_3_	HG vs CON	1.4476	*P* < 0.01	1.5471	up
			HGT vs HG	1.4278	*P* < 0.01	–2.0464	down
4	Lactate	C_3_H_6_O_3_	HG vs CON	1.5497	*P* < 0.05	1.5531	up
			HGT vs HG	1.5763	*P* < 0.05	–3.0531	down
5	Succinates	C_4_H_6_O_4_	HG vs CON	2.1016	*P* < 0.01	–2.1624	down
			HGT vs HG	1.9336	*P* < 0.01	2.1624	up
6	Oxaloacetate	C_4_H_4_O_5_	HG vs CON	2.0293	*P* < 0.05	–1.6257	down
			HGT vs HG	2.1373	*P* < 0.01	1.4355	up
**Amino acids**							
7	Leucine	C_6_H_13_NO_2_	HG vs CON	1.5213	*P* < 0.05	–1.3642	down
			HGT vs HG	1.8203	*P* < 0.01	1.2647	up
8	Valine	C_5_H_11_NO_2_	HG vs CON	1.8042	*P* < 0.05	–1.1261	down
			HGT vs HG	2.1045	*P* < 0.05	1.1152	up
**Biogenic amines**							
9	Putrescine	C_4_H_12_N_2_	HG vs CON	1.3795	*P* < 0.05	1.2485	up
			HGT vs HG	1.9095	*P* < 0.05	–1.6472	down
10	Tyramine	C_8_H_11_NO	HG vs CON	1.4148	*P* < 0.05	1.4471	up
			HGT vs HG	1.4143	*P* < 0.05	–1.5876	down
11	Histamine	C_5_H_9_N_3_	HG vs CON	1.3126	*P* < 0.05	1.5864	up
			HGT vs HG	2.0136	*P* < 0.01	–1.9674	down
**Fatty acid**							
12	Linoleic acid	C_18_H_32_O_2_	HG vs CON	1.7595	*P* < 0.01	–1.5438	down
			HGT vs HG	1.6590	*P* < 0.05	1.2454	up
13	Docosahexaenoic Acid	C_22_H_32_O_2_	HG vs CON	2.1549	*P* < 0.01	–1.3728	down
			HGT vs HG	1.6549	*P* < 0.05	1.7629	up
**Organic acids**							
14	4-Phenylbutyric acid	C_10_H_12_O_2_	HG vs CON	1.6728	*P* < 0.05	–1.0624	down
			HGT vs HG	1.3732	*P* < 0.05	1.0844	up

*^1^CON, basal diets without supplement; HG, high-concentrate diet; HGT, high-concentrate diet supplemented with 200 mg of thiamine / kg of DMI. ^2^VIP, Variable importance in the projection. ^3^FC, Fold change.*

### Correlation Analysis Between Genera and Metabolite Concentrations Affected by the Feeding of Thiamine

**FIGURE 5:**
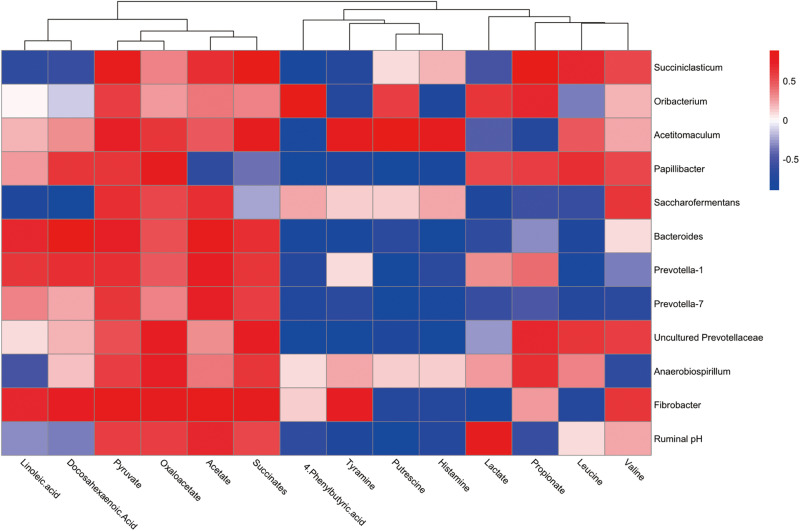
Correlation analysis between genera and metabolite concentrations affected by the feed high-concentrate diet with thiamine supplementation. Each row in the graph represents a genus, each column represents a metabolite, and each lattice represents a Pearson correlation coefficient between a component and a metabolite. Red represents a positive correlation, while blue represents a negative correlation.

To explore the functional correlation between the rumen microbiome changes and metabolite perturbations, a Pearson correlation matrix was generated by calculating the Pearson correlation coefficient in high-concentrate diet with thiamine supplement (Figure 5). The results showed a clear correlation between microbial structure and metabolites (*r* > 0.38 or < 0.38, *P* < 0.05). More interestingly, correlation analysis revealed that acetate was positively associated with eight genera (*r* > 0.49, *P* < 0.05) and negatively associated with one genus (*r* < −0.62, *P* < 0.05). Propionate was positively associated with six genera (*r* > 0.41, *P* < 0.05) and negatively associated with four genera (*r* < −0.55, *P* < 0.05). Pyruvate was positively associated with 12 genera (*r* > 0.52, *P* < 0.05), while lactate was positively associated with three genera (*r* > 0.54, *P* < 0.01) and negatively associated with six genera (*r* < −0.45, *P* < 0.05). Succinates were positively associated with nine genera (*r* > 0.55, *P* < 0.05). Likewise, oxaloacetate was positively associated with nine genera (*r* > 0.47, *P* < 0.05). Leucine was positively associated with four genera (*r* > 0.48, *P* < 0.05) and negatively associated with five genera (*r* < −0.59, *P* < 0.05), whereas valine was positively correlated with five genera (*r* > 0.48, *P* < 0.05) and negatively associated with two genera (*r* < −0.62, *P* < 0.01). Putrescine was positively associated with two genera (*r* > 0.60, *P* < 0.05) and negatively associated with seven genera (*r* < −0.69, *P* < 0.01). Tyramine was positively associated with two genera (*r* > 0.76, *P* < 0.05) and negatively associated with seven genera (*r* < −0.66, *P* < 0.05). Histamine was positively associated with one genus (*r* > 0.78, *P* < 0.05) and negatively associated with eight genera (*r* < −0.65, *P* < 0.05). Linoleic acid was positively associated with three genera (*r* > 0.62, *P* < 0.05) and negatively associated with three genera (*r* < −0.52, *P* < 0.01). Docosahexaenoic acid was positively associated with four genera (*r* > 0.62, *P* < 0.05) and negatively associated with two genera (*r* < −0.57, *P* < 0.05). Finally, 4-phenylbutyric acid (4-PBA) was positively associated with one genus (*r* > 0.86, *P* < 0.01) and negatively associated with seven genera (*r* < −0.64, *P* < 0.05).

## Discussion

### Effects of Thiamine Supplementation on Ruminal Microbial Community Under High-Concentrate Diet Feeding

In the present manuscript, we have demonstrated the alteration of rumen fermentation characteristics by thiamine (Supplementary Table 2), and the data have been published in the *Journal of Dairy Science* (Zhang et al., 2019). To the best of our knowledge, the detailed mechanisms by which thiamine supplementation relieves SARA in goats and dairy cows remain largely unknown. Therefore, to determine the potential mechanism of thiamine on rumen fermentation and bacterial community, we analyzed a high-concentrate diet using 16S rRNA gene sequencing technology.

Our PCA results showed differences in the bacterial community among CON, HG, and HGT groups, indicating the impact of thiamine supplementation. Recent studies have shown that a long-term supply of HG diet results in an alteration of rumen microbial populations (Mao et al., 2015; Hua et al., 2017), while thiamine supplementation in our previous study could increase the abundance of rumen bacterial community (Pan et al., 2017a).

At the phyla level, we observed that high-concentrate dietary supplementation with thiamine might significantly improve the abundance of Bacteroidetes. We also noted that the abundance of Firmicutes decreased in the HGT group compared with the HG group, although the change was statistically insignificant. Of note, the phyla Firmicutes and Bacteroidetes are known for polysaccharide fermentation (Wang et al., 2019). Additionally, Bacteroidetes, one of the most abundant gram-negative bacteria in the lumen, are classified into three categories, namely, Bacteroidetes, *Flavobacteria*, and Sphingobacteria. Zened et al. (2013) reported that an increase in fermentable substrates led to a decrease in pH in the rumen during high-concentrate diet supplementation, which can subsequently lead to death and lysis of gram-negative bacteria (Cole et al., 2005). Firmicutes are predominately composed of gram-positive bacteria (Fernando et al., 2010), which are also insensitive to low pH. Similarly, these bacteria can still degrade easily fermentable carbohydrates under low pH (Wetzels et al., 2015). However, supplementation with thiamine partially reversed this effect.

At the genus level, a high abundance of *Succiniclasticum* was identified in the high-concentrate diet group than in the control group, whereas thiamine supplementation reversed this change, although this result was insignificant. A similar trend was also reported for *Succiniclasticum* with a high-concentrate diet elsewhere (Petri et al., 2013). Importantly, *Succiniclasticum* is an organism that specializes in fermenting succinate by converting it quantitatively to propionate, the most important precursor of glucose in ruminants (Van Gylswyk, 1995). Unfortunately, the function of *Succiniclasticum* in the rumen remains enigmatic. As shown in Supplementary Table 2, the changing trend of propionate content in the rumen was similar to that of *Succiniclasticum*. The low energy yield when anaerobes convert succinate to propionate should make this bacterium highly efficient in this conversion (Thauer et al., 1977). Thiamine is a coenzyme of pyruvate dehydrogenase (PDH) (Chen et al., 2016); the increasing thiamine concentration could facilitate the transformation of pyruvate to acetyl CoA, which ultimately might be hydrolyzed to acetate. The change reduced the production of succinate and subsequently decreased the proportion of *Succiniclasticum* in rumen concentration. *Oribacterium* has been identified as one of the predominant bacteria in the rumen of cows fed forage-based diets (Kong et al., 2010; Kenters et al., 2011), but its role in the rumen remains unknown. Noticeably, thiamine supplementation increased significantly the abundance of *Oribacterium*. However, the reasons for the change in abundance of this genus due to thiamine supplementation need further clarification.

The thiamine treatment group exhibited a higher proportion of the *Anaerobiospirillum*, which can ferment glucose or starch into succinate (Nghiem et al., 1997; Lee et al., 2000). Interestingly, thiamine is associated with the production of acetyl coenzyme A, while acetyl coenzyme A is also related to the production of succinate (Nyström, 2015). The supply of thiamine increased the utilization of the substrate by *Anaerobiospirillum*, hence promoting its proliferation. Besides, Asma et al. (2013) reported the differences due to dietary starch levels in the relative abundances of Fibrobacteres, whose relative abundance decreased by more than two times when the dietary starch level increased. This changing trend is consistent with our results. At present, two species belonging to the genus *Fibrobacter* are known, including *Fibrobacter succinogenes* and *Fibrobacter intestinalis* (Amann et al., 1992). In particular, *F. succinogenes* has been found to utilize its incomplete citric acid cycle to produce succinate, the bacterium’s major fermentative end product (Miller, 1978). Additionally, a recent study has demonstrated that *F. succinogenes* contains both a phosphoenolpyruvate carboxykinase and a pyruvate carboxylase that can reversibly convert pyruvate, respectively, to oxaloacetate, which is sequentially converted to malate, fumarate, and succinate. This degradation process requires thiamine pyrophosphate as a coenzyme factor, which is possibly one of the reasons for the increased content of succinate in the rumen. Moreover, *Prevotella_1*, a polysaccharide-degrading bacteria belonging to the *Prevotella ruminicola* family, in the HGT group tended to be lessened by thiamine supplementation that can promote the transformation of some substances in this degradation pathway (Purushe et al., 2010).

### Effects of Thiamine Supplementation on Ruminal Metabolites

Thiamine contributes significantly to carbohydrate metabolism and is essential for normal cellular function and growth. For the effects of thiamine supplementation on carbohydrate metabolism, our metabolome data indicated that thiamine supplementation alters fermentation type with a high-concentrate diet in the rumen, implying that the ruminal metabolism pattern might be associated with structural changes in bacterial communities. Previous studies have elucidated that supplementation of nonstructural carbohydrates can affect metabolism (Afzal, 1999). As a result, change in carbohydrate structure affects the bacterial communities in the rumen (Mcallan and Smith, 1974). Carbohydrate metabolism plays a vital role in regulating animal health. For example, Sutton (1985) found that the end products of carbohydrates act as energy substrates, inflammation modulators, and signaling molecules in the rumen. As a metabolite of carbohydrates, VFA can regulate the differentiation of the rumen epithelium, cell apoptosis, and morphology of the rumen papilla (Mentschel et al., 2001; Inagaki and Sakata, 2005; Shen et al., 2005). Our findings unearthed that thiamine supplementation in a high-concentrate diet markedly increased the content of acetate and decreased the content of propionate compared with those in the HG group diet in ruminal fluid. The ratio of acetate to propionate is positively correlated with the content of the concentrate (Lechartier and Peyraud, 2010). Studies have emphasized that the succinate pathway is the principal pathway of propionate production, which is used by Bacteroidetes to generate propionate from a substrate (Louis et al., 2014). More importantly, this may be a plausible explanation for the decrease in the ratio of acetate to propionate with thiamine supplementation relative to the high-concentrate diet group. Besides, as a coenzyme factor of PDH (Bubber et al., 2004), deficiency of thiamine leads to the accumulation of pyruvate, which is then converted to lactate under the catalytic action of lactate dehydrogenase (LDH) (Kumar et al., 2015). The supplement of thiamine can promote the conversion of pyruvate into acetyl-CoA, reducing the accumulation of lactate and pyruvate. Moreover, thiamine is a coenzyme required for the activity of α-ketoglutarate dehydrogenase (α-KGDHC) that is involved in the Krebs cycle (Hanson and Cox, 1967). The increase in oxaloacetate content can be interpreted as the enhancement of the tricarboxylic acid cycle, which promotes the transformation of oxaloacetate.

For amino acid metabolism, amino acids play a pivotal role in the rumen, whereby rumen microbes are associated with amino acid metabolism (Chalupa, 1975). Our data demonstrate that the content of leucine and valine in the rumen was considerably increased by thiamine supplementation in the high-concentrate diet. Amino acids are the degradation products of microbial or dietary proteins and precursors of protein synthesis in the rumen, which can regulate some metabolic pathways (Mariz et al., 2018). Previous studies have shown that L-leucine tended to decrease the level of acetate in the rumen (Menahan and Schultz, 1964). This result concurs with our findings. The relationship between leucine and thiamine has been rarely reported in the rumen. Thiamine supplementation has been noted to promote the transformation of acetyl-CoA, which is key to amino acid metabolism (Schwartau et al., 1984). Furthermore, TPP, as a coenzyme factor of hydroxyl acetate synthase, catalyzes the first step reaction to the biosynthesis of valine (Byrne, 1998), while thiamine supplementation can enhance this metabolic pathway.

For biogenic amine metabolism, high concentrations of bioamines such as histamine, putrescine, and tyramine may induce a series of inflammatory responses in the rumen epithelium (Nocek, 1997; Fusi et al., 2004), which emerges in the rumen because of decarboxylation of precursor amino acids under the degradation of ruminal microbes (Wang et al., 2013). Studies have suggested that the absorption of histamine in the rumen increases the content of histamine in the whole organism accompanied by the damage of the rumen epithelial cells caused by lactate poisoning, which is the main factor that aggravates the condition of ruminal acidosis (Andersen and Jarlv, 1990). In this work, we found that thiamine supplementation with a high-concentrate diet significantly decreased the content of histamine, putrescine, and tyramine. It has been postulated that gram-negative bacteria die and lyse when carbohydrates ferment quickly and pH drops sharply, leading to the release and absorption of LPS and histamine (Duffield et al., 2004; Khafipour et al., 2009). The stimulation of toll-like receptor 4 (TLR4) by LPS induces the release of critical proinflammatory cytokines (Lu et al., 2008), while thiamine might regulate the pathways (Pan et al., 2017b). Besides, competitive inhibition of histamine and thiamine have been reported in the study of bacterial metabolism (Terzl and Keek, 1949), which is likely one of the reasons that thiamine supplementation reduces biogenic amines.

For fatty acid (FA) metabolism, diet composition can modify the content and composition of ruminal bacterial FAs (Bas and Archimède, 2003). Rumen microbes are assumed to play an essential role in the metabolism of trans-FA and conjugated linoleic acid (Kalscheur et al., 1997). Also, a shift in the rumen bacteria community has been reported as dietary NDF content (Dehority and Orpin, 1997). This could have influenced FA composition due to the large differences in FA composition in different community bacterial structures (Minato et al., 1988). In this study, the contents of linoleic acid and docosahexaenoic acid in the rumen were decreased significantly with thiamine supplementation in a high-concentrate diet. A reasonable explanation for FA alteration may be that thiamine supplement perhaps changed the bacterial community structure (Pan et al., 2017a). Moreover, thiamine is a coenzyme factor in the synthesis of acetyl-CoA, which is a precursor for the synthesis of FA; thus, thiamine supplementation can facilitate this pathway.

For organic acid metabolism, the 4-PBA plays an integral role in maintaining proteostasis and apoptosis of cells (Sun et al., 2012; Kolb et al., 2015). Studies have revealed that the 4-PBA reduced cellular damage as well as cell death (Sawada et al., 2008) and attenuated inflammation (Qi et al., 2011). This may be an indication that thiamine supplementation increased the content of 4-PBA, thus alleviating rumen epithelial inflammation. Currently, there are few reports on the association between 4-PBA and bacterial community structure in animals. It is also important to note that the underlying mechanism by which thiamine supplementation affects the metabolism of 4-PBA requires further investigation.

### Correlations Between Bacterial Community and Metabolism With Thiamine Supplementation

The relationship between bacterial community and metabolism with thiamine supplementation has been discussed above. In general, there was a utilization or productive association between bacteria and metabolomes in the rumen throughout the study. Mao et al. (2015) concluded that the type of feeding affects the concentration of metabolites by influencing bacterial community structure, hence affecting the rumen function. Taken together, these changes and correlations provide new insights that can reveal the mechanism of action of thiamine on animal health.

## Conclusion

We applied Illumina sequencing and metabolomics analysis to reveal thiamine modulation of ruminal microbiota and metabolites in goats fed a high-concentrate diet in this study. As a result, thiamine supplementation of a high-concentrate diet may modify the rumen fermentation, which is partially attributed to the growing abundances of *Oribacterium*, *Anaerobiospirillum*, *Fibrobacter*, and uncultured *Prevotellaceae*, as well as the decreasing proportions of *Prevotella_1*. We also found that thiamine supplementation might help alleviate high-grain-induced SARA by modifying bacterial community structure, resulting in changes in the metabolism of carbohydrates, amino acids, FAs, biogenic amines, and organic acids. Overall, our results shed light on understanding the function of thiamine on ruminal metabolism modulation and also provide new guidelines to alleviate SARA.

## Data Availability Statement

The datasets presented in this study can be found in online repositories. The names of the repository/repositories and accession number(s) can be found below: PRJNA700131.

## Ethics Statement

All management and experimental procedures were conducted according to the Animal Protection Law based on the Guidelines for the Care and Use of Laboratory Animals approved by the Ethics Committee of Yangzhou University (SXXY 2015-0054).

## Author Contributions

YM, HZ, and HW designed the research. CW, LY, and LD conducted the research. DG, JY, and HZ analyzed the data. YM wrote the manuscript. YM and CW had the primary responsibility for the final content. All authors read and approved the final manuscript.

## Conflict of Interest

The authors declare that the research was conducted in the absence of any commercial or financial relationships that could be construed as a potential conflict of interest.
