# Supplementary figures and images for: Illumina Sequencing and Metabolomics Analysis Reveal Thiamine Modulation of Ruminal Microbiota and Metabolome Characteristics in Goats Fed a High-Concentrate Diet
Source: Front Microbiol. 2021 Apr 7;12:653283. doi: 10.3389/fmicb.2021.653283 (PMC8058204; doi:10.3389/fmicb.2021.653283)

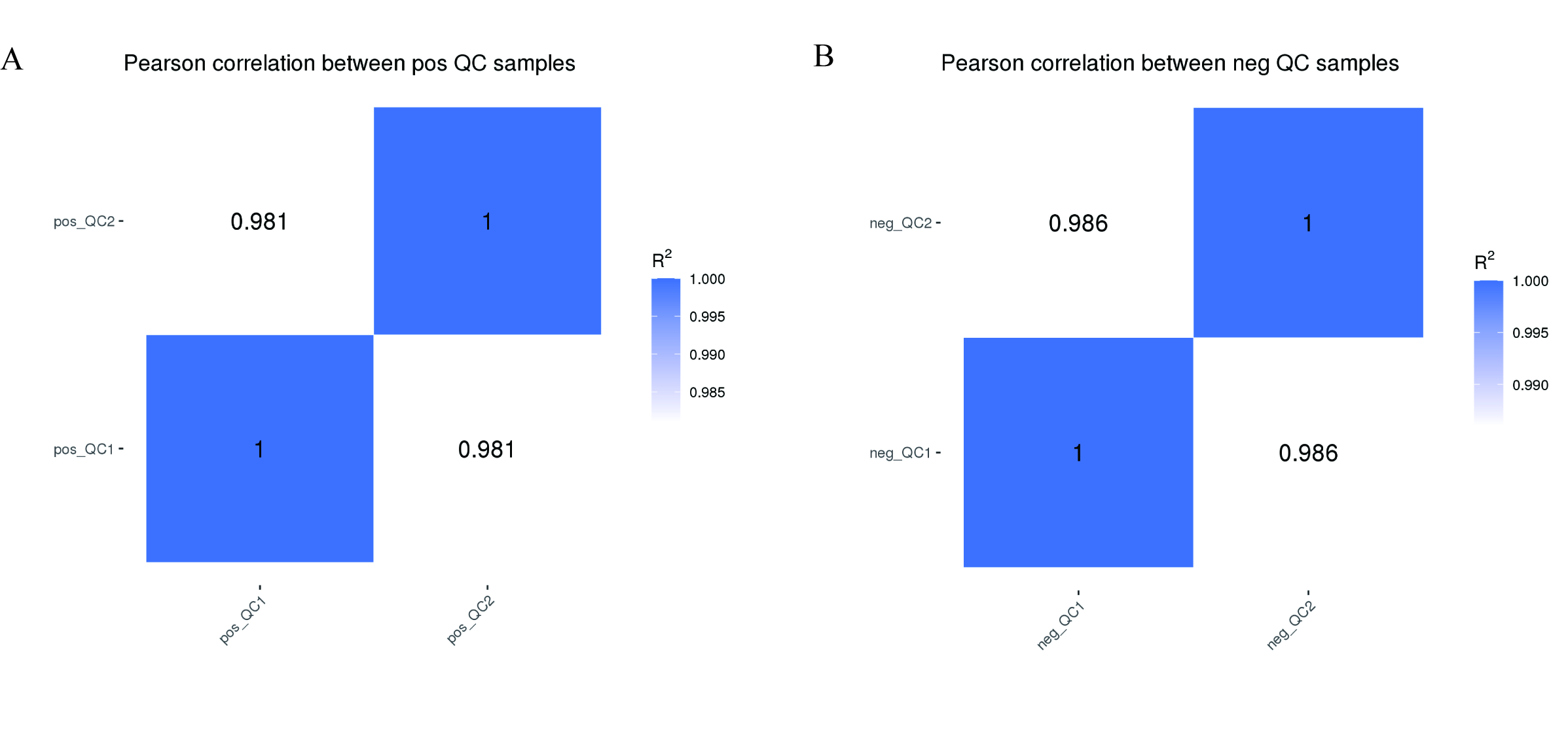

Supplement: Supplementary file 1 [file Image_1.TIF]

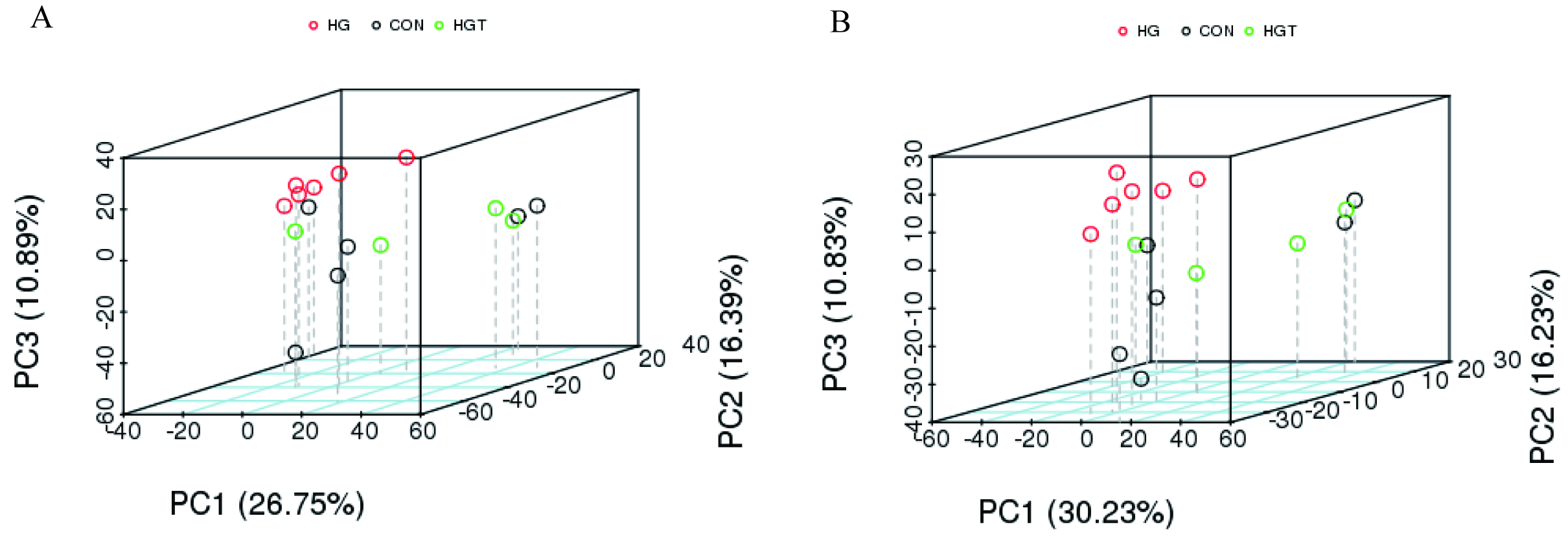

Supplement: Supplementary file 2 [file Image_2.TIF]
