# Supplementary material for: Illumina Sequencing and Metabolomics Analysis Reveal Thiamine Modulation of Ruminal Microbiota and Metabolome Characteristics in Goats Fed a High-Concentrate Diet
Source: Front Microbiol. 2021 Apr 7;12:653283. doi: 10.3389/fmicb.2021.653283 (PMC8058204; doi:10.3389/fmicb.2021.653283)
Supplement: Supplementary file 3 [file Table_1.DOCX]

**Table S1**. Ingredients and nutritional composition of diets^1^ offered to lactating Saanen goats

| Item | CON | HG | HGT |
| --- | --- | --- | --- |
| Ingredient (% of DM) |  |  |  |
| Chinese wildrye | 70.00 | 30.00 | 30.00 |
| Corn grain | 14.00 | 58.90 | 58.90 |
| Soybean meal | 13.00 | 8.45 | 8.45 |
| Calcium hydrophosphate | 1.42 | 0.53 | 0.53 |
| Limestone | 0.58 | 1.12 | 1.12 |
| Salt | 0.50 | 0.50 | 0.50 |
| Premix^2^ | 0.50 | 0.50 | 0.50 |
| Nutrient composition |  |  |  |
| ME (MJ/kg of DM) | 8.81 | 11.72 | 11.72 |
| CP (% of DM) | 10.81 | 10.79 | 10.79 |
| NDF (% of DM) | 44.28 | 26.71 | 26.71 |
| ADF (% of DM) | 23.89 | 13.27 | 13.27 |
| Starch (% of DM) | 23.16 | 48.38 | 48.38 |
| Calcium (% of DM) | 0.81 | 0.78 | 0.78 |
| Phosphorus (% of DM) | 0.47 | 0.42 | 0.42 |
| Thiamine (mg/kg of DM) | 1.20 | 1.90 | 201.90 |

^1^CON = control; HG = high-concentrate diet; HGT = highconcentrate diet supplemented with 200 mg of thiamine/kg of DMI.

^2^Premix consisted of the following ingredients per kilogram of diet: 6.00 × 10^3^ IU of vitamin A, 3.0 × 10^3^ IU of vitamin D, 82.0 mg of vitamin E, 6.15 mg of Cu, 70.0 mg of Fe, 65.0 mg of Zn, 47.0 mg of Mn, 0.135 mg of I, 0.115 mg of Co, and 0.115 mg of Mo.

**Table S2**. Effects of thiamine supplementation on metabolites in the rumen of Saanen goats with SARA

| Item^1^ | Diet^2^ | | | SEM | *P*-value |
| --- | --- | --- | --- | --- | --- |
|  | CON | HG | HGT |  |  |
| Ruminal variables |  |  |  |  |  |
| pH | 6.11^a^ | 5.42^b^ | 6.03^a^ | 0.11 | 0.003 |
| Thiamine (µg/L) | 7.15^a^ | 2.08^c^ | 4.02^b^ | 0.18 | 0.006 |
| Acetate (mM) | 38.98^a^ | 27.32^b^ | 37.21^a^ | 2.08 | 0.007 |
| Propionate (mM) | 15.76^c^ | 31.61^a^ | 21.08^b^ | 1.99 | 0.005 |
| Butyrate (mM) | 8.53^c^ | 19.39^a^ | 12.08^b^ | 0.87 | 0.008 |
| Isobutyrate (mM) | 1.12 | 1.36 | 1.28 | 0.12 | 0.078 |
| Valerate (mM) | 0.42^c^ | 0.88^a^ | 0.64^b^ | 0.04 | 0.003 |
| Isovalerate (mM) | 0.41^c^ | 0.92^a^ | 0.69^b^ | 0.07 | 0.006 |
| Total VFA (mM) | 65.22^c^ | 81.48^a^ | 72.98^b^ | 2.09 | 0.009 |
| Acetate: propionate ratio | 2.47^a^ | 0.86^c^ | 1.77^b^ | 0.10 | 0.007 |
| Lactate (mM) | 0.34^c^ | 0.71^a^ | 0.52^b^ | 0.04 | 0.003 |
| Pyruvate (mM) | 0.21^c^ | 0.33^a^ | 0.26^b^ | 0.02 | 0.012 |
| PDH (IU/L) | 3.12^a^ | 1.03^c^ | 2.38^b^ | 0.11 | 0.009 |
| Free LPS (×10^3^ EU/mL) | 25.09^c^ | 55.87^a^ | 42.16^b^ | 3.08 | 0.011 |
| NH3-N (mM) | 24.17^a^ | 11.28^c^ | 17.89^b^ | 1.21 | 0.006 |

^a–c^Within a row, means without a common letter differ (P < 0.05).

^1^PDH = pyruvate dehydrogenase.

^2^CON = control; HG = high-concentrate diet; HGT = high-concentrate diet supplemented with 200 mg of thiamine/kg of DMI. n = 6 goats/group.
